# Medical and Physical Intelligence

**Published:** 1804-01-01

**Authors:** 


### MEDICAL AND PHYSICAL INTELLIGENCE.

[ FOREIGN AND DOMESTIC. ]

In the Medical Journal, published at Montpelier, we find two cases of children poisoned by the use of bitter almonds ; in one, the person had eaten them heated in a copper vessel ; in the other, the child had been made to drink the milk of bitter almonds as a remedy against worms.

It is rather singular, the variety of opinions that exist on the pernicious effects of these bodies ; some maintaining them to be harmless when used by man, while they are acknowledged to be highly deleterious to other animals, particularly birds. The learned Prof. Murray made many experiments on different animals, which prove their poisonous qualities, but in different degrees in different animals. In those animals, the subjects of his experiments, as well ai in the cases of the children, the effects of drunkenness were very remarkable. On the other hand, Fred. Hoffman asserts, Medic. Ration. System. vol. 1. that their bad effects are but but little observed in the human subject, but, like nux vomica, highly so to other animals; he instances the dog, cat, pigeon, &c. The experiments of Schrader, a distinguished chemist of Berlin, are remarkable. He found that the prussic acid was contained in laurel water, in the infusion of peach leaves, in bitter almonds, as well as many other vegetables; and desirous to ascertain if this acid produced the same effects on the animal economy as the distilled laurel water, bitter almonds, &c. he gave a sparrow some drops of it, which was immediately killed. The same author asserts, that birds respiring the vapours of prussic acid, die as quickly as those exposed to the smell of laurel water. If bitter almonds contain prussic acid, and if this acid be mortal, may we not conelude that their pernicious effects depend on the prussic acid which they contain?

---

Professor Hufeland, first physician to his Prussian Majesty, at Berlin, highly praises the external use of *flores zinci* in excoriations and cutaneous ulcerations, aud has employed them with great success in painful excoriations of the nipples, in ulcerated herpes, and in the obstinate remains of an inveterate itch. This remedy diminishes the pains, is exsiccant, and healing within a short time, without being attended with any danger, which we have always to dread from the external use of lead and its preparations. He generally makes use of the following unguent: ℞. Ungt. pomat. unc. j. flor. zinci semin. lycopod. aa dr. ß. M. This ointment he orders to be applied three times a day, spread on pieces of soft linen. He relates a case of a child, one year old, which had suffered from its birth an herpetic eruption, which grew so bad, that the child was covered all over with sores, attended with much pain, by which it was entirely emaciated. Antimonials, mercurials, baths, lime water, and a solution of sublimate, externally applied, were used without any avail, and even seemed to increase the complaints. At last he applied the ungt. flor. zinc, and had the satisfaction to see this remedy mitigate the pains, and heal the sores, so that within a fortnight no ulcerating place remained, and the child at present enjoys the best health.

---

The same gentleman recommends the following tincture of digitalis purpurea, to which he gives the name of tinctura digitalis aquoso ætherea, and in which all the efficacious particles of this plant are concentrated. Take three ounces of very good digitalis, well dried and cut in small pieces, macerate them with eighteen ounces of distilled water for twenty-four hours, now and then shaking the vessel, and then strain, pouring on the herb distilled water, till it passes off without colour and taste. Evaporate the liquor that is thus obtained in the water bath, till three ounces remain. The herb that has been in this manner extracted, being carefully dried, is infused with six ounces of spiritus sulphurico

ætheréus

ætherens (liquor anodynus) and after the space of twenty-four hours it is expressed, strained, and added to those three ounces of liquor. This tincture contains the extractive, resinous, albuminous and acrid matter of that medicinal plant.

---

Mr. Kelch of Königsburg, has made some remarkable Galvanic experiments on the body of a criminal who was beheaded for a capital crime. The pile of which he made use consisted of sixty-two plates of zinc and copper, which was combined with another of fifty-two strata. The head immediately after the execution was placed on a table, and while the spinal marrow, which was cut through at the sixth vertebra colli, was touched with the conductor of the zinc pole, the conductor of the copper pole was applied to the left upper eye-lid, and immediately the eyes, which were only half shut, opened themselves, in which state they remained as long as the chain was shut. The eye-lids not only contracted themselves, but shewed a tremulous motion, which ceased immediately after removing the conductors. The contractions were still stronger, on moistening the eye-lids with a solution of sal ammon. No change could be produced either in the iris or in the pupilla. On touching the ala nasi and the septum mobile with the copper pole conductor, the ala distended itself and became tremulous, the septum and the point of the nose were drawn downwards. By touching the middle part of the upper lip it approached to the under lip, but not so much as to shut the half-open mouth. On applying the conductor of the copper-pole to the corner of the mouth, the upper lip was contracted, while the under lip remained immovable; the tongue shewed undulating motions when touched with the conductor of the copper pole. Similar contractions followed in the temples and cheeks. On touching the spinal marrow, and any part of the face, the fore part of the neck came into a sudden motion, resembling the act of swallowing, which lasted some time after having removed the conductor. All these experiments were several times repeated, and continued for above half an hour. On the left arm, a place two inches large of the musculus biceps was laid bare, and having touched the spinal marrow with the conductor of the zinc pole, and that place with the copper pole conductor, the muscle was suddenly contracted; the fore arm turned itself, moving at the same time towards the body, and likewise the upper arm made a turning motion, drawing near the body; but as soon as the conductor was removed, the arm fell back into its former situation. On bringing the spinal marrow and the scrobiculus cordis within the Galvanic chain, the latter part, with all the integuments of the belly, began to raise itself; the thorax contracted, forming a convexity; the arms became stiff, were raised and moved towards the trunk; the shoulders were lifted; the upper part of the spine was bent, moving somewhat down the table; but when the Galvanic chain was opened, all these motions disappeared.

peared. On repeating the experiment, the same motions ensued, except the latter. The muscles of the belly likewise showed contractions. The large and small intestines could by no means be excited to contract, nor yet the stomach, though they had previously been moistened with a solution of sal ammon. While the zinc pole remained in combination with the spinal marrow, the conductor of the copper pole was applied to the abdominal surface of the diaphragm, and to the processus ensiformis of the sternum, by which it was considerably contracted, but a motion of the heart could not be perceived. Touching the pericardium produced not any contraction; but on opening it, and touching the heart on the anterior part of the right ventricle, some slight motions at the apex cordis, and where the large blood-vessels enter the ventricles, and at the right auricula, could be produced. The heart was taken out of the body, and being laid on the hand of a gentleman who assisted at the experiments, the zinc conductor was introduced into the right ventricle, while the apex cordis and the surface of the left ventricle was touched with the copper conductor, by which means considerable contractions, and particularly an alternate contraction and distension of the orifice of the right ventricle, where the arteries enter it, and of its auricle were produced, which even continued for a few minutes after having removed the conductors. The limbs having become cool during the former experiments showed but slight contractions. On laying bare and galvanising the musculus Sartorius, the motions of the thigh were more considerable. A cutaneous branch of the crural nerve, touching with its inner surface the musculus sartorius was prepared and combined with a bare place of the muscle of the other limb; the nerve contracted, while slight motions were at the same time perceived in that muscle, to which the zinc conductor had been applied, and the place of the nerve which had been touched with the conductor became the next day light brown, and dry, while the remaining part was soft, moist, and of its natural colour.

---

### St. Thomas's and Guy's Hospitals.

The Spring Course of Lectures will commence as usual in the beginning of February, viz.

Anatomy and Operations of Surgery, by Mr. Cline and Mr. Cooper. — Practice of Medicine, by Dr. Babington and Dr. Curry. — Chemistry and Experimental Philosophy, by Dr. Babington and Mr. Allen. — Theory of Medicine and Materia Medica, by Dr. Curry. — Midwifery, and Diseases of Women and Children, by Dr. Haighton. — Physiology, or Laws of the Animal Œconomy, by Dr. Haighton. — Principles and Practice of Surgery, by Mr. Cooper.

Sometime in the Spring, Mr. Coleman, Professor at the Veterinary College, will give a Course of Lectures on the Veterinary Art.

These Lectures are so arranged, that no two of them interfere in the hour of attendance; and the whole is calculated to form a complete Course of Medical and Surgical Instruction.—Terms and other particulars to be learnt of Mr. STOCKER, Apothecary to Guy's Hospital.

---

THEATRE of ANATOMY, Blenheim Street, Great Marlborough Street.

Mr. BROOKES will commence his Spring Course of Lectures on Anatomy, Physiology, and Surgery, on Saturday the 21st of January, 1804, at two o'clock.—The Dissecting Rooms are open from eight in the morning until two, where Mr. Brookes attends.

---

Dr. CLARKE's Spring Course of Lectures on Midwifery and the Diseases of Women and Children, will begin on Monday, January 30, at a quarter past ten o'clock, at the house of Dr. Clarke, No. 1, New Burlington Street. These Lectures are not given in the summer.

---

Dr. BATTY's Lectures on Midwifery, and the Diseases of Women and Children, will begin on Monday, Jan. 30, at half past ten o'clock, at his house in Great Marlborough Street.

---

Mr. TAUNTON's Spring Course of Lectures on Anatomy, Physiology, and Surgery, will recommence on the 21st day of January. 1804, at eight o'clock in the evening; and the Lectures will be continued every Tuesday, Thursday, and Saturday, at the same hours. Particulars may be known by application to Mr. Taunton, No. 10, Paternoster Row.

---

Mr. SYER, Successor to Dr. Pole, will commence his Second Course of Winter Lectures on the Elements and Practice of Midwifery, at his Lecture Room, 102, Leadenhall Street, on Monday, Jan. 8, 1804, at six o'clock in the evening.

---

Dr. KISBY, of Edinburgh, proposes this Winter, or in the ensuing Spring, to deliver a Course of Lectures on the Principles and Practice of Medicine.

